# How Different Electrolytes Can Influence the Aqueous Solution Behavior of 1-Ethyl-3-Methylimidazolium Chloride: A Volumetric, Viscometric, and Infrared Spectroscopy Approach

**DOI:** 10.3389/fchem.2020.593786

**Published:** 2020-11-12

**Authors:** Shokat Sarmad, Mohammed Taghi Zafarani-Moattar, Dariush Nikjoo, Jyri-Pekka Mikkola

**Affiliations:** ^1^Technical Chemistry, Department of Chemistry, Chemical-Biological Centre, Umeå University, Umeå, Sweden; ^2^Physical Chemistry Department, University of Tabriz, Tabriz, Iran; ^3^Division of Material Science, Luleå University of Technology, Luleå, Sweden; ^4^Industrial Chemistry & Reaction Engineering, John Gadolin Process Chemistry Centre, Åbo Akademi University, Turku, Finland

**Keywords:** ionic liquids, 1-ethyl-3-methyl-imidazolium chloride, apparent molar volume, apparent isentropic compressibility, viscosity *B*-coefficient

## Abstract

The density, sound velocity, and viscosity of 1-ethyl-3-methylimidazolium chloride [C_2_mim]Cl in pure water and aqueous solutions of some electrolytes such as potassium chloride, potassium carbonate, and potassium phosphate (weight fraction of salt fixed at *w*_s_ = 0. 11) have been measured over a wide range of temperatures from 298.15 to 318.15 K. The obtained experimental data have been used to compute various volumetric, compressibility, and viscometric parameters, e.g., apparent molar properties, limiting apparent molar and transfer properties. The co-sphere overlap model was employed to describe the dominant intermolecular interactions in the ternary solutions. Additionally, the structure making/breaking nature of the [C_2_mim]Cl in the ternary solutions has been discussed in terms of Hepler's constant and the temperature derivative of viscosity *B*-coefficient (*dB*/*dT*). The activation free energy of solvent and solute, activation enthalpy, and activation entropy have been calculated by the application of transition state theory. The calculated parameters have been interpreted in the sense of solvent–solute and solute–solute interactions. The Fourier transform infrared (FTIR) studies also have been done for the studied systems. Volumetric, acoustic, viscometric, and spectroscopic studies can render some evidence and help to understand the aqueous solution behavior of ionic liquids.

## Introduction

Ionic liquids (ILs) have been utilized and studied in the last decades in different fields like organic catalysis, biomass treatment/conversion, gas separation, electrolytes in the extraction of diverse substances, and reaction media, due to their inherent advantages and unique properties such as negligible vapor pressure, high thermal and chemical stability, designable ability, and excellent solubility (Wang et al., 2014; Sarmad et al., 2017; Cao et al., 2018; Xu et al., 2018; Hui et al., 2019; An et al., 2020; Wu et al., 2020).

They are considered as potential environmentally friendly solvents to replace the common volatile organic solvents and thus reduce environmental footprints (Gomes et al., 2019; Kaur et al., 2020). In industrial applications, instead of pure compounds, frequently, mixtures of ILs with different solvents are used, and therefore, investigation of the behavior of binary or ternary mixtures of ILs is of great importance to unravel their structural characteristics which can provide valuable information regarding solute–solute and solute–solvent interactions (Zafarani-Moattar and Sarmad, 2012).

Imidazolium-based ILs have been gaining more attention in the field of materials, physics, and chemistry as pure solvents or cosolvents in aqueous/non-aqueous systems and biphasic mixtures (Wang et al., 2007; Zafarani-Moattar and Sarmad, 2011). Most of the studies have been related to the synthesis of ILs and their chemical reactions. Indeed, investigation of the physicochemical properties of the systems containing ILs in a wide range of pressures and temperatures is in demand. These properties are essential for optimizing and designing the systems containing IL and solvent in both laboratory and industrial scales. Additionally, for the evaluation of molecular interactions, thermophysical properties and their deviations from ideality are important (Pal and Gaba, 2008).

Kaur et al. studied volumetric and acoustic properties of choline acetate IL in α, ω-alkane diols, at different temperatures (Kaur et al., 2020). Volumetric, acoustic, and spectroscopic properties of an aqueous ternary mixture of IL 1-hexyl-3-methylimidazolium bromide and amino acids (l-alanine and l-phenylalanine) were investigated by Kumar and Sharma (2020). Volumetric, acoustic, and infrared spectroscopic study of amino acids in aqueous solutions of pyrrolidinium-based IL was also carried out by Kumar et al. (2020). Sharma et al. (2020) studied molecular interactions of l-histidine in aqueous imidazolium-based IL solutions using volumetric, acoustic, and viscometric approaches, at different temperatures. On the other hand, Zafarani-Moattar et al. (2020) investigated the volumetric, acoustic, and viscometric properties of some choline amino acid ILs in aqueous polypropylene glycol 400 and polyethylene glycol 400 solutions.

Marcinkowski et al. (2019) studied interactions of *N*-alkyl-*N*-methylmorpholinium-based ILs with acetonitrile using molecular dynamic simulations along with density and velocity of sound measurements.

The volumetric and acoustic behaviors of a protic IL, 2-hydroxy ethylammonium lactate, was investigated in different hydroxylic media, e.g., water, methanol, and ethanol, at various temperatures (288.15–323.15 K) and under atmospheric pressure by Barros et al. (2018). The influence of temperature, alkyl chain length, and nature of the anion on the volumetric and acoustic properties of imidazolium-based ILs with alkyl nitriles was studied as well (Bhanuprakash et al., 2020).

Interactions of 1-butyl-1-methylpyrrolidinium tetrafluoroborate and 1-butyl-1-methyl piperidinium tetrafluoroborate with acetonitrile and water were investigated by density and speed of sound measurements comparatively and at a different temperature, by Sahin and Ayranci (2019). Gaba et al. (2019) studied the molecular interactions of l-glutamic acid and l-aspartic acid in aqueous imidazolium-based IL solutions, at different temperatures, by measuring their volumetric and acoustic properties. Furthermore, the apparent molar volume, apparent molar isentropic compressibility, and corresponding limiting values were computed using the experimental density and speed of sound data. These quantities are helpful when investigating the solvation behavior and various interactions present in the ternary amino acid + IL + water solutions.

To understand the intermolecular interaction existing in the ternary systems of citrate salts + water + 1-butyl-3-methyl imidazolium tetrafluoroborate, Kumar and Katal (2018b) studied the volumetric and compressibility properties at 0.1 MPa and different temperatures. The structure-making ability of citrate salts in IL solution is observed.

Zafarani and Sarmad investigated the effect of tripotassium phosphate, dipotassium hydrogen phosphate, and potassium dihydrogen phosphate on aqueous solution behavior of 1-ethyl-3-methylimidazolium bromide at different temperatures. The negative *dB*/*dT* values of IL in water and aqueous electrolyte solutions revealed that IL acts as a structure maker in water and in aqueous electrolyte solutions (Zafarani-Moattar and Sarmad, 2010).

Volumetric, acoustic, and transport properties of IL, 1-butyl-3-methyl imidazolium chloride in aqueous lithium bromide solutions at different temperatures (298.15–318.15 K) were studied as well. The calculated volumetric interaction parameters indicated the existence of hydrophilic–ionic or ionic–ionic interactions in the studied systems and suggested that the interactions between the IL and lithium bromide are mainly pairwise (Zafarani-Moattar et al., 2014).

The interactions of some phosphate and citrate salts with 1-hexyl-3-methylimidazolium chloride as a function of temperature have been investigated by a combination of volumetric and acoustic measurements by Kumar and coworkers. The absorption values revealed the existence of interactions between citrate/phosphate salt, IL, and water molecules, which supported results obtained from volumetric and acoustic data (Kumar and Chadha, 2014, 2015).

Volumetric and compressibility behaviors of binary and ternary aqueous solutions of 1-hexyl-3-methylimidazolium chloride in methyl potassium malonate and methyl potassium malonate were studied by Sadeghi and Mahdavi (2012); the obtained results have been interpreted in terms of the solute–solute and solute–water interactions.

Ternary systems composed of phosphate salts + 1-butyl-3-methyl imidazolium tetrafluoroborate + water and citrate salts + 1-butyl-3-methyl imidazolium tetrafluoroborate + water at 0.1 MPa and different temperature have been investigated by Kumar and Katal (2018a,c). The pair and triplet interaction coefficients have been computed from transfer parameters and have demonstrated the supremacy of pairwise interactions in the system. The possible intermolecular interactions such as hydrophilic–hydrophobic, hydrophilic–hydrophilic, or hydrophobic–hydrophobic were investigated as well.

In the present study, density, speed of sound, and viscosity of 1-ethyl-3-methyl imidazolium chloride [C_2_mim]Cl in pure water as well as in an aqueous solution of potassium chloride, potassium carbonate, and potassium phosphate (*w*_*s*_ = 0.11) at *T* = 298.15–318.15 K were measured. The apparent molar volume, isentropic compressibility, apparent molar isentropic compressibility, and relative viscosity were computed using the experimental data. Transfer apparent molar volume, apparent isentropic compressibility, and viscosity *B*-coefficient of IL from pure water to aqueous electrolyte solutions were also calculated which provides valuable information regarding the solute–solute and solute–solvent interactions that exist in aqueous IL + electrolyte solutions. The structure-making/breaking nature of the [C_2_mim]Cl in the ternary solutions has been discussed in terms of Hepler's constant and temperature derivative of viscosity *B*-coefficient (*dB*/*dT*). The activation free energy, enthalpy, and entropy were also calculated by the application of transition state theory. The calculated parameters were interpreted in the sense of solvent–solute and solute–solute interactions. The Fourier transform infrared (FTIR) studies and thermal analysis were also performed for the studied systems. The temperature dependency of viscosity was successfully fitted to the Vogel–Fulcher–Tammann (VFT) equation.

## Materials and Methods

All chemicals (electrolytes and IL) were obtained from Merck with a purity >98 (wt%). The electrolytes were used as received without further purification, but they were dried at 100–110°C overnight. The water content in the IL was determined using a microprocessor-based automatic Karl–Fischer titrator. The water content found for [C_2_mim]Cl was 1.16% by mass. This water content in the IL was taken into account during the preparation of the aqueous solutions of IL. For the preparation of solutions, double-distilled and deionized water was used.

All the solutions were prepared afresh and scaled using an analytical balance (Shimadzu, 321-34553, Shimadzu Co., Japan) with precision (10^−7^ kg). All the solutions were kept tightly sealed to minimize the absorption of atmospheric moisture. Measurements were performed immediately after the preparation of solutions.

### Density Measurements

The density and sound velocity of mixtures were measured using a commercial density and sound velocity measurement apparatus (Anton Paar DSA 5000 densimeter and sound velocity analyzer). Both of the speed of sound and density are extremely sensitive to temperature, and therefore, the temperature was kept constant within ±10^−3^ K. The experimental uncertainties of density and ultrasonic velocity measurements were estimated to be in the range of ±3.0 ± 10^−6^ g·cm^−3^ and ±0.1 m·s^−1^, respectively. The apparatus was calibrated with double-distilled deionized and degassed water and dry air at atmospheric pressure.

### Viscosity Measurements

The viscosity measurements were carried out with a suspended level Ubbelohde-type capillary viscometer, mounted in a water thermostat (Julabo, MD-18V, Germany) which was calibrated with water at five different temperatures (298.15–318.15 K). The flow time of a constant volume of liquid through a capillary was measured with an electronic stopwatch with a resolution of 0.01 s. An average of at least four readings of flow time with a variation not exceeding ±0.1 s was calculated for each solution. The temperature of the thermostat bath was controlled within ±0.01 K. The measured viscosities were found to be accurate within ±0.001 mPa. Also, the dynamic viscosity, η, was calculated by applying the following relation:

(1) $$\begin{array}{l} \eta =dK\left(t-\theta \right) \end{array}$$

here, *t, K*, θ, and *d* are referred to as the flow time, the viscometer constant, the Hagenbach correction factor, and the density, respectively. The viscometer constant was determined by calibrating with distilled water at working temperatures (Marsh, 1987). The uncertainty for the dynamic viscosity determination was estimated to be around ±0.5%.

### Thermogravimetric Analysis (TGA)

STA 449C Jupiter (NETZSCH, Germany) was employed to perform the TGA. Five to 10 mg of samples was heated under argon atmosphere from 20 to 500°C, at a heating rate of 10°C·min^−1^. NETZSCH TA software was used for the processing of the obtained data.

### Fourier Transform Infrared (FTIR) Analysis

The attenuated total reflectance Fourier transforms infrared spectroscopy (ATR-FTIR) was utilized to characterize the [C_2_mim]Cl in pure water and aqueous electrolyte solutions. The Bruker Vertex 80v FTIR spectrometer with a DTGS detector was applied. The FTIR analysis was performed at room temperature for all the studied systems.

## Results and Discussions

### Volumetric Properties

The experimental density and sound velocity for binary aqueous [C_2_mim]Cl solution along with ternary [C_2_mim]Cl + aqueous electrolyte solution at *T* = 298.15–318.15 K are reported in Supplementary Table 1. Apparent molar volumes, *V*_ϕ_, of [C_2_mim]Cl in pure water and aqueous electrolyte solutions (*w*_*s*_ = 0.11), at different temperatures, were determined using the experimental density data via the following equation:

(2) $$\begin{array}{l} V_{\varphi }=\frac{\left(\rho_{0}-\rho \right)}{\rho }+\frac{M}{\rho_{0}} \end{array}$$

where *M* and *m* are the molar mass (kg·mol^−1^) and the molality (mol·kg^−1^) of [C_2_mim]Cl, respectively; ρ and ρ_0_ are the densities (kg·m^−3^) of ternary solutions ([C_2_mim]Cl + H_2_O + electrolyte) and the solvent (H_2_O + electrolyte), respectively. The apparent molar volume in the aqueous electrolyte solutions is larger than that of pure water and increases by increasing the temperature. This means that the size of the solute [C_2_mim]Cl in aqueous electrolyte solutions is larger than in pure water. This can be attributed to the phenomena that in aqueous solutions, [C_2_mim]Cl dissociated to anion and cation, whereas in the presence of potassium salts, they formed ion pairs with the ions of the salts which are physically bonded together and, therefore, the electrostatic interactions between water molecules and the ions decrease. In the studied electrolyte solutions, the apparent molar volume decreased by increasing the charge of anions as follows: KCl > K_2_CO_3_ > K_3_PO_4_.

The limiting apparent molar volumes, at infinite dilution ($V_{\varphi }^{0}$), were estimated by applying the Redlich–Mayer-type equation to the corresponding data via Equation (3) (Redlich and Meyer, 1964).

(3) $$\begin{array}{l} V_{\varphi }=V_{\varphi }^{0}+S_{V}m^{\frac{1}{2}}+b_{V}m \end{array}$$

**Figure 1:**
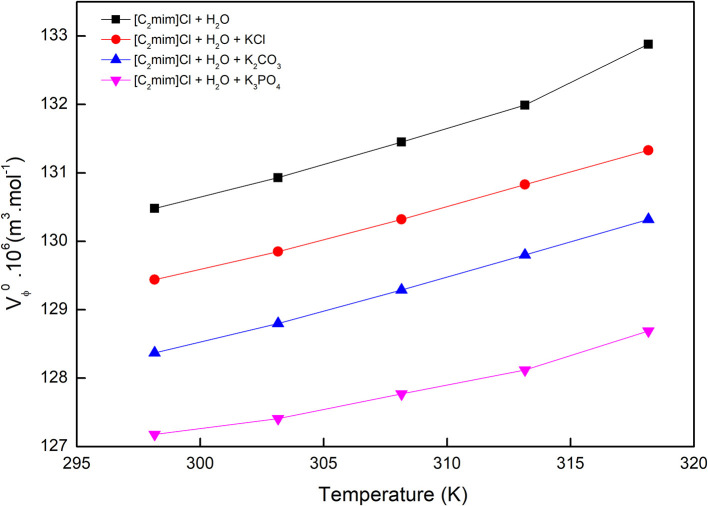
Limiting apparent molar volumes of studied systems at different temperatures.

The $V_{\varphi }^{0}$ values along with adjustable parameters (*S*_V_, *b*_V_) and standard deviations are summarized in Table 1. The magnitude of $V_{\phi }^{0}$ values increases with an increase in temperature as can be depicted in Figure 1, indicating that the hydration effects in solutions are strongly sensitive to temperature. The limiting apparent molar volumes render beneficial information regarding solvent–solute interactions. The reason is that at infinite dilution, each ion is surrounded just by solvent molecules and is distant with other ions. Consequently, the limiting apparent molar volume is a measure of ion–solvent interaction and uninfluenced by the ion–ion interactions. The calculated limiting apparent molar volumes were used to determine the limiting molar volumes of transfer of IL from water to aqueous electrolyte solutions (Rajagopal and Jayabalakrishnan, 2009; Zafarani-Moattar and Sarmad, 2010; Kaczkowska et al., 2020).

(4) $$\begin{array}{l} \Delta_{t}V_{\varphi }^{0}=V_{\varphi }^{0}\left(\text{in aq}.\text{ electrolyte solution}\right)-V_{\varphi }^{0}\left(\text{in pure water}\right) \end{array}$$

**Table 1:** Limiting apparent molar volumes along with the coefficients of Equation (3) for the studied systems.

***T* (K)**	**$10^{6}\times V_{\varphi }^{0}$ (m^3^·mol^**−1**^)**	**10^**6**^ × *S*_**v**_ (m^**3**^·kg^**1/2**^·mol^**−3/2**^)**	**10^**6**^ × *b*_**v**_ (m^**3**^·kg·mol^**−2**^)**	**sd**
**[C**_**2**_**mim]Cl** **+** **H**_**2**_**O**				
298.15	130.48	−0.06	−1.03	0.01
303.15	130.93	−0.06	−0.93	0.01
308.15	131.45	−0.04	−0.98	0.01
313.15	131.99	−0.01	−1.10	0.01
318.15	132.88	−0.01	−1.71	0.01
**[C**_**2**_**mim]Cl** **+** **H**_**2**_**O** **+** **KCl (*****w**_***s***_* **=** **0.11)**				
298.15	129.44	−0.07	−5.25	0.04
303.15	129.85	0.34	−5.48	0.04
308.15	130.32	0.06	−5.36	0.04
313.15	130.83	0.18	−5.44	0.06
318.15	131.33	−0.02	−5.33	0.05
**[C**_**2**_**mim]Cl** **+** **H**_**2**_**O** **+** **K**_**2**_**CO**_**3**_ **(*****w**_***s***_* **=** **0.11)**				
298.15	128.37	0.05	−5.56	0.06
303.15	128.80	0.39	−5.91	0.05
308.15	129.29	0.79	−6.32	0.05
313.15	129.80	0.27	−5.86	0.03
318.15	130.32	0.05	−5.80	0.05
**[C**_**2**_**mim]Cl** **+** **H**_**2**_**O** **+** **K**_**3**_**PO**_**4**_ **(*****w**_***s***_* **=** **0.11)**				
298.15	127.18	−0.18	−6.08	0.03
303.15	127.41	0.32	−6.39	0.03
308.15	127.77	0.37	−6.41	0.05
313.15	128.12	0.36	−6.35	0.05
318.15	128.69	−0.38	−5.89	0.05

**Figure 2:**
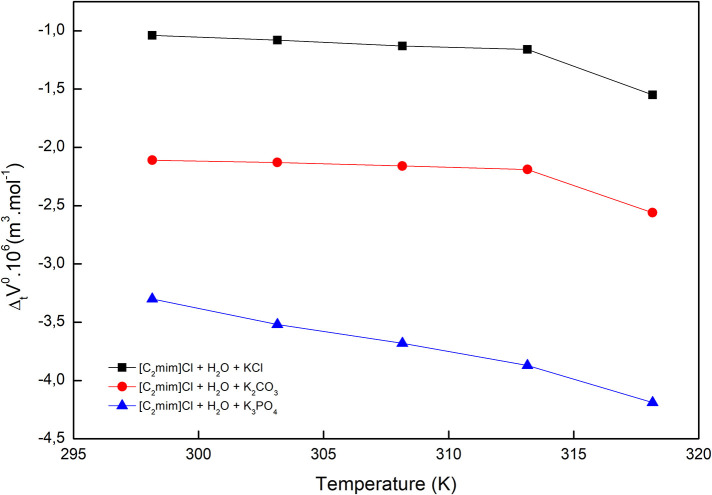
Limiting apparent molar volumes of transfer of [C_2_mim]Cl from pure water to aqueous electrolyte solutions.

The obtained values $\Delta_{t}V_{\varphi }^{0}$ are reported in Table 2 and depicted in Figure 2. Evidently, as can be seen for all studied systems, the values are negative and generally decrease with increasing temperature. The negative values suggest that the hydrophobic–ionic interactions predominate over the hydrophilic–ionic interactions.

**Table 2:** Limiting molar volume, limiting molar isentropic compressibility, and *B*-coefficient of transfer of [C_2_mim]Cl from pure water to aqueous electrolyte solutions (*w*_*s*_ = 0.11).

		***T*****/*****K***				
	**System**	**298.15**	**303.15**	**308.15**	**313.15**	**318.15**
$\Delta_{t}V_{\varphi }^{0}$	[C_2_mim]Cl + H_2_O + KCl	−1.04	−1.08	−1.13	−1.16	−1.55
	[C_2_mim]Cl + H_2_O + K_2_CO_3_	−2.11	−2.13	−2.16	−2.19	−2.56
	[C_2_mim]Cl + H_2_O + K_3_PO_4_	−3.30	−3.52	−3.68	−3.87	−4.19
$\Delta_{t}K_{\varphi }^{0}$	[C_2_mim]Cl + H_2_O + KCl	0.93	0.91	0.78	0.75	0.61
	[C_2_mim]Cl + H_2_O + K_2_CO_3_	1.16	1.02	0.98	0.77	0.68
	[C_2_mim]Cl + H_2_O + K_3_PO_4_	1.09	1.01	0.87	0.66	0.47
Δ_*t*_*B*	[C_2_mim]Cl + H_2_O + KCl	0.113	0.117	0.106	0.104	0.089
	[C_2_mim]Cl + H_2_O + K_2_CO_3_	0.153	0.152	0.136	0.134	0.119
	[C_2_mim]Cl + H_2_O + K_3_PO_4_	0.190	0.181	0.167	0.161	0.146

**Scheme 1:**
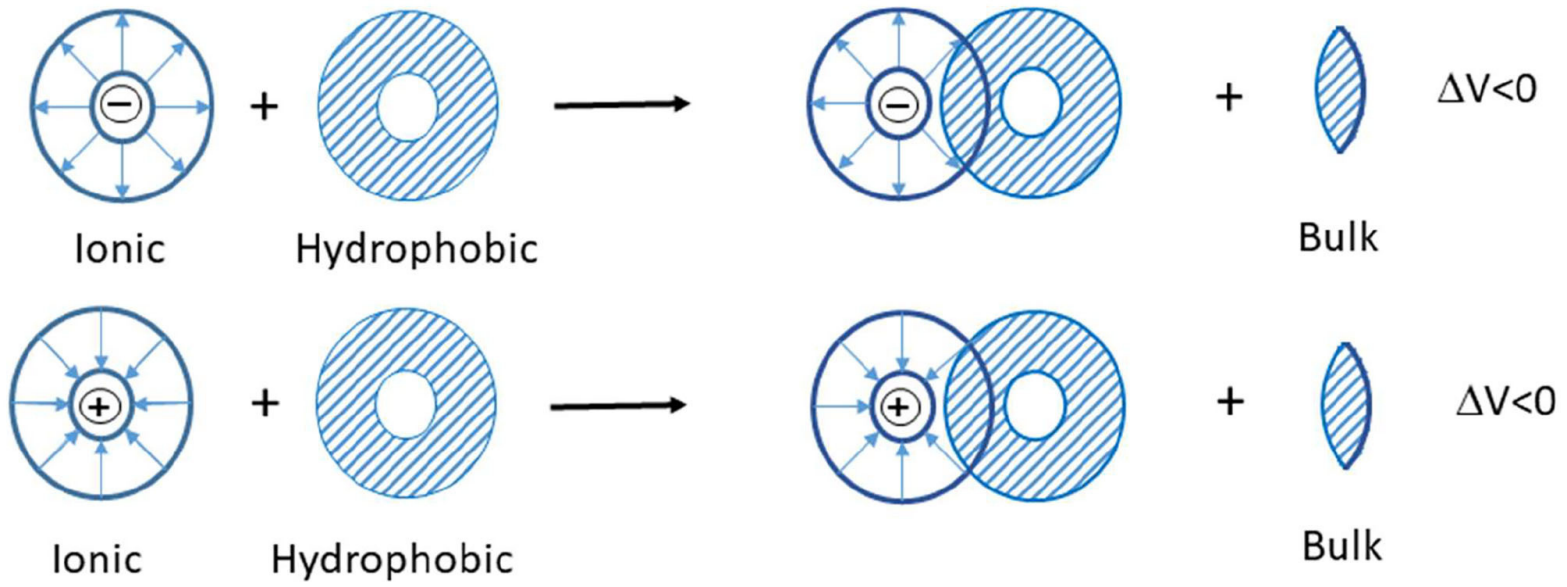
The interaction of two co-spheres (Lin et al., 2006).

In the studied ternary system ([C_2_mim]Cl + H_2_O + electrolyte), three types of interactions possibly exist between the solvent and solute: (1) ion–ion interactions among potassium ions of electrolytes and chloride ions of IL, (2) ion–ion interactions among imidazolium ion of IL and phosphate, carbonate, and chloride ions of electrolytes, (3) ion–apolar group interactions. In the systems in which type 3 interactions are dominant, the limiting molar volumes of transfer are negative. According to the co-sphere overlap model, the overlap of two co-spheres of hydrophobic and ionic hydration releases some water molecule from the solvation sphere to the bulk that leads to a negative volume contribution (Scheme 1) (Friedman and Krishnan, 1973; Lin et al., 2006).

To identify the hydrophilic or hydrophobic features of [C_2_mim]Cl in electrolyte solutions, the temperature dependency of the obtained $V_{\varphi }^{0}$ values was correlated via the following quadratic equation (Rajagopal and Jayabalakrishnan, 2009):

(5) $$\begin{array}{l} V_{\varphi }^{0}=a+bT+cT^{2} \end{array}$$

here *a, b*, and *c* are empirical coefficients that have been determined so Equation (5) has the following form for the studied systems.

(6) $$\begin{array}{r} V_{\varphi }^{0}=339.476-1.468\text{T}+0.003{\text{T}}^{2}\left(\text{for aqueous solution}\right)\\ V_{\varphi }^{0}=160.674-0.292\text{T}+0.001{\text{T}}^{2}\left(\text{for KCl }{\text{w}}_{\text{s}}=\;0.11\right)\\ V_{\varphi }^{0}=153.325-0.254\text{T}+0.001{\text{T}}^{2}\left(\text{for }{\text{K}}_{2}\text{C}{\text{O}}_{3}\;{\text{w}}_{\text{s}}=\;0.11\right)\\ V_{\varphi }^{0}=286.524-1.105\text{T}+0.002{\text{T}}^{2}\left(\text{for }{\text{K}}_{3}\text{P}{\text{O}}_{4}\;{\text{w}}_{\text{s}}=0.11\right) \end{array}$$

According to Hepler's theory, Hepler's constant ${\left(\frac{\partial^{2}V_{\varphi }^{0}}{\partial T^{2}}\right)}_{P}$ is related to the hydrophilic or hydrophobic feature of the solute and is a better proof in describing whether a solute acts as a breaker or maker of a certain structure (Helper, 1969). If the sign of Hepler's constant is positive, it means that the solute is hydrophobic and is behaving as making (developing) the structure. *Vice versa*, the negative sign shows that the solute is hydrophilic and acts as a breaker of the structure. The obtained ${\left(\frac{\partial^{2}V_{\varphi }^{0}}{\partial T^{2}}\right)}_{P}$ values for all the studied systems are positive (Equation 6) and revealed that [C_2_mim]Cl in pure water and electrolyte solutions is a structure maker.

Differentiating Equation 5 to temperature results in the limiting apparent molar expansibility ($E_{\varphi }^{0}$).

(7) $$\begin{array}{l} E_{\varphi }^{0}={\left(\frac{\partial V_{\varphi }^{0}}{\partial T}\right)}_{P}=b+2cT \end{array}$$

The obtained values of $E_{\varphi }^{0}$ for studied systems are reported in Table 3. At all temperatures, $E_{\varphi }^{0}\;$ values of IL in water and electrolyte solutions are positive and increased with temperature due to the increase of volume. This behavior can be attributed to the solvation and shrinkage of the solvent around the ions in aqueous electrolyte solutions. Some of the water molecules upon heating the solution may be released from the hydration layer, which can increase the volume of solution slightly faster than that of pure water, so $E_{\varphi }^{0}$ will be positive. Under ambient conditions, the expansivity of water is around 0.25 × 10^−3^ K^−1^, which is less than that in aqueous IL solutions (Gu and Brennecke, 2002).

**Table 3:** Limiting apparent molar expansibility and isobaric thermal expansion coefficients for studied systems ([C_2_mim]Cl + H_2_O + electrolyte; w_s_ = 0.11) at different temperatures.

		***T*****/*****K***				
	**System**	**298.15**	**303.15**	**308.15**	**313.15**	**318.15**
$10^{6}\times E_{\phi }^{0}$ (m^3^·mol^−1^·K^−1^)	[C_2_mim]Cl + H_2_O	0.049	0.124	0.199	0.274	0.349
	[C_2_mim]Cl + H_2_O + KCl	0.083	0.089	0.095	0.101	0.108
	[C_2_mim]Cl + H_2_O + K_2_CO_3_	0.087	0.092	0.098	0.104	0.109
	[C_2_mim]Cl + H_2_O + K_3_PO_4_	0.036	0.055	0.075	0.094	0.113
10^3^× α_*P*_ (K^−1^)	[C_2_mim]Cl + H_2_O	0.65	0.70	0.74	0.79	0.84
	[C_2_mim]Cl + H_2_O + KCl	0.38	0.95	1.51	2.08	2.63
	[C_2_mim]Cl + H_2_O + K_2_CO_3_	0.68	0.71	0.76	0.80	0.84
	[C_2_mim]Cl + H_2_O + K_3_PO_4_	0.28	0.42	0.58	0.72	0.86

The limiting apparent molar expansibility along with limiting apparent molar volume was used to determine the isobaric thermal expansion coefficient (α_*P*_) through Equation (8).

(8) $$\begin{array}{l} \alpha_{P}=\frac{1}{V_{\varphi }^{0}}{\left(\frac{\partial V_{\varphi }^{0}}{\partial T}\right)}_{P}=\frac{E_{\varphi }^{0}}{V_{\varphi }^{0}} \end{array}$$

The obtained values of α_*P*_ are listed in Table 3. The value of α_*P*_ in water was higher than that in electrolyte solutions.

### Acoustic Properties

The isentropic compressibility was determined using the experimental density and speed of sound by applying Laplace–Newton's equation.

(9) $$\begin{array}{l} \kappa_{s}=\frac{1}{\rho u^{2}} \end{array}$$

**Figure 3:**
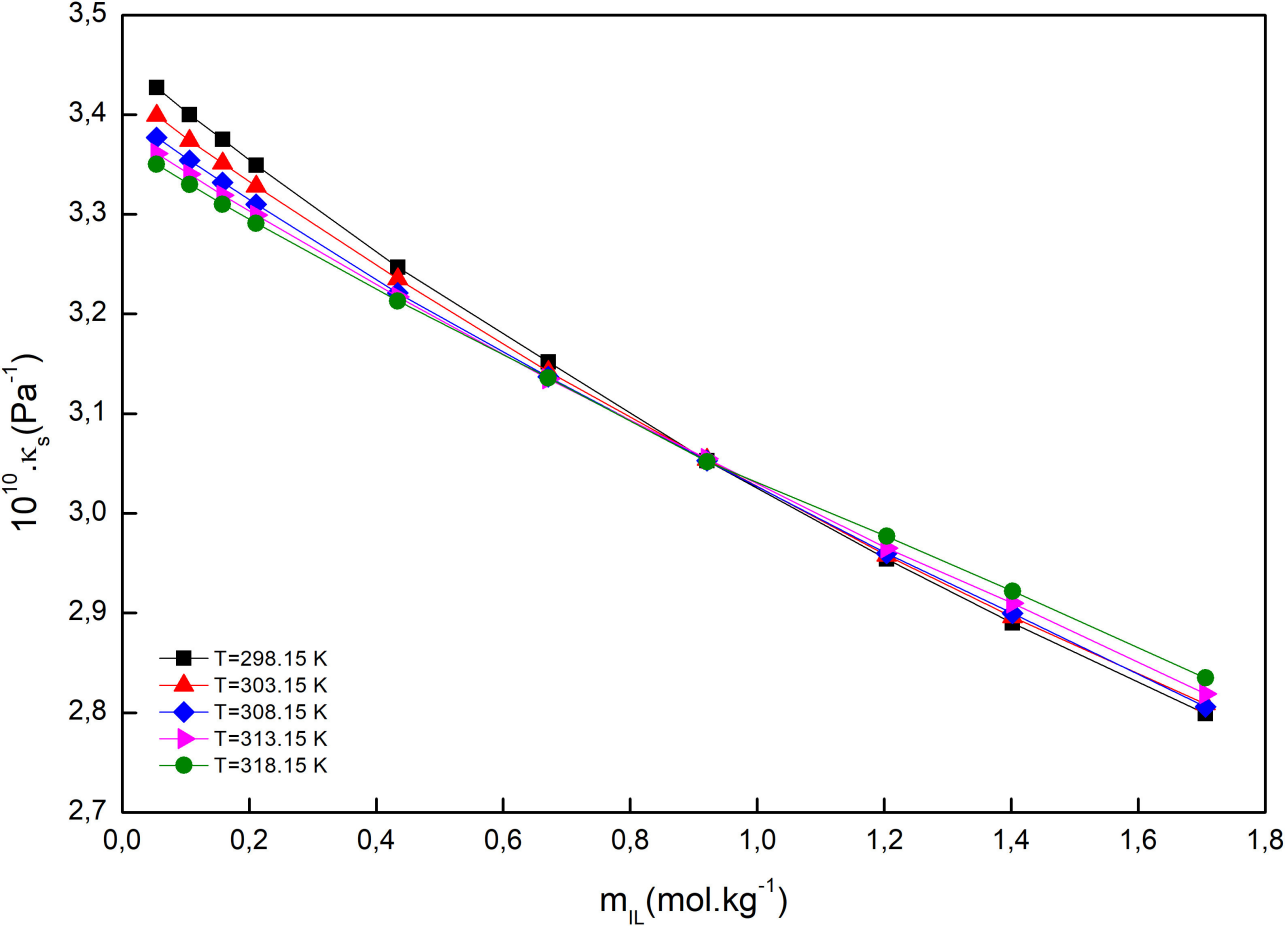
Isentropic compressibility of [C_2_mim]Cl + H_2_O + K_3_PO_4_ as a function of [C_2_mim]Cl molality at different temperatures.

For all the studied systems, the isentropic compressibility decreased by the molality of IL, at all studied temperatures, and the reason may be hydration of ions and breaking up of the three-dimensional network of water molecules. The compressibility isotherms for the system [C_2_mim]Cl + H_2_O + K_3_PO_4_, as an example, is demonstrated in Figure 3. As can be seen, the isotherms intersect at *m*_IL_ = 0.921 mol·kg^−1^; at this point, the isentropic compressibility was equal and temperature independent. The same behavior has been observed in the electrolyte and non-electrolyte solutions (Schmelzer et al., 2004; Wahab and Mahiuddin, 2004).

According to Equation (10), the isentropic compressibility of electrolyte solutions is the sum of two contributions (Wahab and Mahiuddin, 2004):

(10) $$\begin{array}{l} \kappa_{\text{s}}\left(\text{electrolyte solution}\right)=\kappa_{\text{s}}\left(\text{solvent intrinsic}\right)\\ \;+\kappa_{\text{s}}\left(\text{solute intrinsic}\right) \end{array}$$

We can see (Figure 3) that up to the converging point, κ_*s*_ (solvent intrinsic) is the main contributor to the total value of isentropic compressibility, and after that, the solute intrinsic part is the major contributor. At the coverage point, *d*κ_*s*_/*dT* = 0, and it seems that at this point the inherent structure of water is totally broken down, and therefore, the water molecules are assimilated in the ions' primary hydration shell. Above the coverage point, *d*κ_*s*_/*dT* > 0, so the contribution of the solvent intrinsic properties is almost naught, in contrast to the case when we are below the coverage point, *d*κ_*s*_/*dT* < 0. Indeed, the presence of an intersection point can be attributed to the formation of a clathrate-like structure in an aqueous IL solution similar to tetraethyl ammonium salts, which exhibited a similar behavior (Jerie et al., 1999).

The apparent molar isentropic compressibility of the studied systems was computed by employing Equation (11).

(11) $$\begin{array}{l} K_{\varphi }=\frac{\left(\kappa_{s}\rho_{0}-\kappa_{s0}\rho \right)}{m\rho \rho_{0}}+\frac{\kappa_{s}M}{\rho } \end{array}$$

Here, κ_*s*0_ and κ_*s*_, respectively, represent the isentropic compressibility of a pure solvent and a mixture. Since the concentration dependency of the apparent molar isentropic compressibility was non-linear, the obtained values were fitted to the Redlich–Mayer-type equation as below:

(12) $$\begin{array}{l} K_{\varphi }=K_{\varphi }^{0}+S_{K}m^{\frac{1}{2}}+\;b_{K}m \end{array}$$

The adjustable parameters of the above equation along with limiting apparent isentropic compressibility, at infinite dilution ($K_{\varphi }^{0}$), are tabulated in Table 4. The obtained values for [C_2_mim]Cl in aqueous solutions, at all temperatures, were negative and smaller than those obtained for the electrolyte solutions. In the studied electrolyte solutions, the $K_{\varphi }^{0}$ values at lower temperatures are negative, which means that the water molecules around IL presented higher resistances to compression when compared with bulk. Also, the values of $K_{\varphi }^{0}$ increased by increasing the temperature. The negative values at lower temperatures can be attributed to the attractive interactions between the IL and solvent due to the hydration of the ions. The values of $K_{\varphi }^{0}$ are affected by the size of the molecules and the degree of penetration of solvent molecules. The size of the molecules has a positive contribution to $K_{\varphi }^{0}\;$, and thereby, large molecules due to the free intermolecular spaces possess inherent compressibility more so than in the case of a mixture. The penetration of the solvent molecules has a negative contribution; this can result in the formation of intramolecular free spaces because the IL molecules interacted with the solvent molecules. Upon increasing temperature, the electrostriction was reduced, and the interactions of IL with solvent were weakened. Thus, the solvent molecules around the IL are released into the bulk; and therefore, the medium becomes more compressible, and the value of $K_{\varphi }^{0}$ is increased.

**Table 4:** Limiting apparent molar isentropic compressibility along with the coefficients of Equation (6) for the studied systems at different temperatures.

***T* (K)**	**10^**14**^·$K_{\varphi }^{0}$ (m^**3**^·mol^**−1**^·Pa^**−1**^)**	**10^**14**^·*S*_**k**_ (m^**3**^·mol^**−1**^·Pa^**−1**^)**	**10^**14**^·*b*_**k**_ (m^**3**^·mol^**−1**^·Pa^**−1**^)**	**10^**14**^·σ**
**[C**_**2**_**mim]Cl** **+** **H**_**2**_**O**				
298.15	−1.72	0.98	−0.20	0.01
303.15	−1.34	1.28	−0.52	0.01
308.15	−0.93	1.33	−0.65	0.02
313.15	−0.51	1.16	−0.57	0.01
318.15	−0.12	0.91	−0.42	0.01
**[C**_**2**_**mim]Cl** **+** **H**_**2**_**O** **+** **KCl (*****w***_**s**_ **=** **0.11)**				
298.15	−0.79	0.77	−0.34	0.01
303.15	−0.41	0.65	−0.32	0.01
308.15	−0.15	0.64	−0.31	0.01
313.15	0.24	0.37	−0.19	0.01
318.15	0.49	0.45	−0.22	0.01
**[C**_**2**_**mim]Cl** **+** **H**_**2**_**O** **+** **K**_**2**_**CO**_**3**_ **(*****w***_**s**_ **=** **0.11)**				
298.15	−0.56	1.29	−0.66	0.03
303.15	−0.36	1.55	−0.88	0.03
308.15	0.09	1.18	−0.74	0.02
313.15	0.26	1.23	−0.79	0.01
318.15	0.56	1.05	−0.71	0.01
**[C**_**2**_**mim]Cl** **+** **H**_**2**_**O** **+** **K**_**3**_**PO**_**4**_ **(*****w**_***s***_* **=** **0.11)**				
298.15	−0.63	0.78	−0.35	0.02
303.15	−0.33	0.86	−0.47	0.01
308.15	−0.06	0.90	−0.57	0.01
313.15	0.15	1.04	−0.70	0.01
318.15	0.35	1.01	−0.69	0.01

The limiting molar compressions of transfer ($\Delta K_{\varphi }^{0}$) from water to aqueous electrolyte solutions have been determined using the following relation:

(13) $$\begin{array}{l} \Delta_{\text{t}}{\text{K}}_{\varphi }^{0}={\text{K}}_{\varphi }^{0}\left(\text{in aq}.\text{ electrolyte solution}\right)-{\text{K}}_{\varphi }^{0}\left(\text{in pure water}\right) \end{array}$$

The obtained values are listed in Table 2, and for all studied systems, the values of $\Delta K_{\varphi }^{0}\;$ are positive and decrease with increasing temperature.

### Viscosity

The relative viscosities of [C_2_mim]Cl in aqueous and electrolyte solutions were determined using the experimental viscosity data (Supplementary Table 2) and employing the following equation.

(14) $$\begin{array}{l} \eta_{r}=\frac{\eta }{\eta_{0}} \end{array}$$

Herein, η and η_0_ are the viscosities of the solution and solvent, respectively. The viscosity *B*-coefficients were obtained by fitting the relative viscosity (in the linear region) to the Jones–Dole equation by a least-squares method (Jones and Dole, 1929).

(15) $$\begin{array}{l} \eta_{r}=\frac{\eta }{\eta_{0}}=1+BC \end{array}$$

In the above equation, *C* is the molarity of solutions (mol·dm^−3^). The obtained *B*-coefficients are presented in Table 5. For all the studied systems, *B*-coefficients are positive and decreased by increasing temperature as well as decreasing the charge of the anion (KCl < K_2_CO_3_ < K_3_PO_4_).

**Table 5:** Viscosity *B*-coefficients for the studied systems at different temperatures.

***T*/K**	***B*/(dm^**3**^·mol^**−1**^)**	**σ (η)**
**[C**_**2**_**mim]Cl** **+** **H**_**2**_**O**		
298.15	0.2883	0.004
303.15	0.2805	0.004
308.15	0.2773	0.004
313.15	0.2738	0.003
318.15	0.2793	0.002
**[C**_**2**_**mim]Cl** **+** **H**_**2**_**O** **+** **KCl (*****w**_***s***_* **=** **0.11)**		
298.15	0.4017	0.029
303.15	0.3978	0.020
308.15	0.3833	0.020
313.15	0.3780	0.016
318.15	0.3682	0.015
**[C**_**2**_**mim]Cl** **+** **H**_**2**_**O** **+** **K**_**2**_**CO**_**3**_ **(*****w**_***s***_* **=** **0.11)**		
298.15	0.4410	0.045
303.15	0.4327	0.038
308.15	0.4137	0.031
313.15	0.4077	0.029
318.15	0.3986	0.025
**[C**_**2**_**mim]Cl** **+** **H**_**2**_**O** **+** **K**_**3**_**PO**_**4**_ **(*****w**_***s***_* **=** **0.11)**		
298.15	0.4783	0.062
303.15	0.4616	0.055
308.15	0.4439	0.046
313.15	0.4349	0.038
318.15	0.4251	0.035

The viscosity *B*-coefficients are of great importance in the investigation of the transport properties of solutions. They render valuable information regarding the solvation of the solute and their influence in the structure of the solvent surrounding the solute molecules. Additionally, some activation parameters can be determined using *B*-coefficients. The viscosity *B*-coefficients depend on the size of the solvent and solute molecules as well as the interactions between the solvent–solute (Jenkins and Marcus, 1995; Rajagopal and Jayabalakrishnan, 2009). Positive and large *B*-coefficient values demonstrate a structure-making phenomenon, thus rendering the hydrophobicity of the solute molecules and giving rise to hydrogen-bonded action of solute on solvent molecules. Table 5 illustrates the structure-making ability of [C_2_mim]Cl in both aqueous and electrolyte solutions, and the values reflect the presence of strong ion–solvent interactions. The temperature derivatives of the *B*-coefficients (*dB*/*dT*) of the studied systems are negative since their values decrease by increasing temperature. The sign of *dB*/*dT* also provides some information regarding the structure-making/breaking ability of a solute in a solvent media (Sarma and Ahluwalia, 1973). For [C_2_mim]Cl in aqueous and electrolyte solutions, the values of *dB*/*dT* are negative and indicate the structure-making feature of [C_2_mim]Cl. These results are in excellent agreement with those concluded from Hepler's constant.

The viscosity *B*-coefficients were used to calculate Δ_*t*_*B* transfer as follows:

(16) $$\begin{array}{l} \Delta_{t}B=B-\text{coefficient }\left(\text{in aq}.\text{ electrolyte solution}\right)\\ \;-B-\text{coefficient }\left(\text{in pure water}\right) \end{array}$$

**Figure 4:**
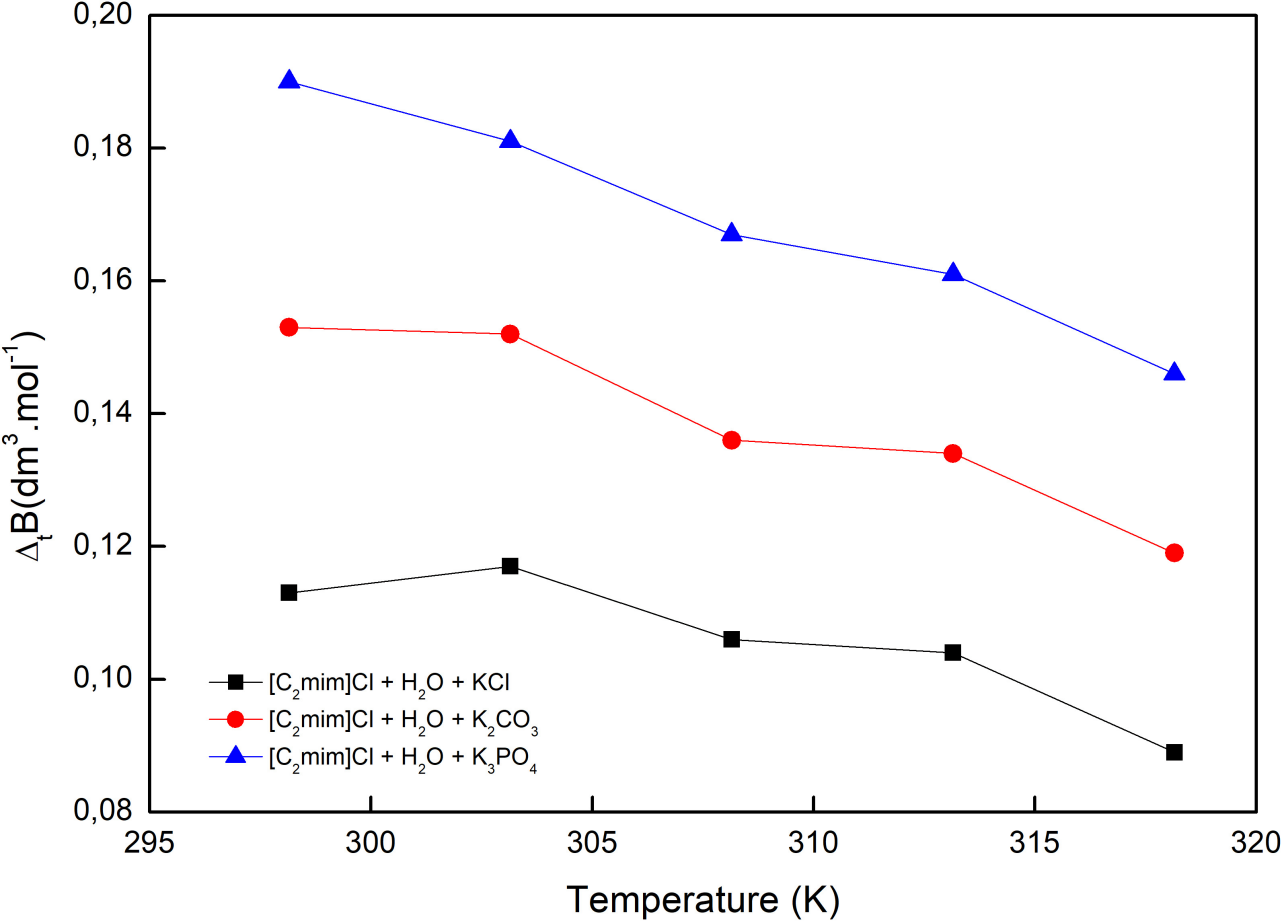
Viscosity *B*-coefficient of transfer of [C_2_mim]Cl from pure water to aqueous electrolyte solutions.

The reported values of Δ_*t*_*B* in Table 2 and Figure 4, for all cases, are positive and increase by increasing the temperature as well as decreasing the charge of electrolytes' anion. The positive values of Δ_*t*_*B* represented the more structured medium in the presence of aqueous electrolyte solutions.

The viscosity *B*-coefficients were also used to evaluate the free energy of activation per moles of the solvent and solute by employing the transition state theory suggested by Feakins et al. (1993) and Glasstone et al. (1941):

(17) $$\begin{array}{l} B=\frac{V_{\varphi ,1}^{0}-V_{\varphi ,2}^{0}}{1,000}+\frac{V_{1}^{0}\left(\Delta \mu_{2}^{0*}-\Delta \mu_{1}^{0*}\right)}{1,000RT} \end{array}$$

(18) $$\begin{array}{l} \Delta \mu_{1}^{0*}=RT\ln\left(\frac{\eta_{0}{V\varphi ,}_{1}^{0}}{hN_{A}}\right) \end{array}$$

(19) $$\begin{array}{l} \Delta \mu_{2}^{0*}=\Delta \mu_{1}^{0*}+\frac{RT\left[1000B-\left(V_{\varphi ,1}^{0}-V_{\varphi ,2}^{0}\right)\right]}{V_{\varphi ,1}^{0}} \end{array}$$

Here, $V_{\varphi ,1}^{0}$ and $V_{\varphi ,2}^{0}$ are the limiting apparent molar volumes of the solute and solvent, respectively. $\Delta \mu_{1}^{0*}$ and $\Delta \mu_{2}^{0*}$ denote the activation free energies of the solute and solvent, respectively. *h* is Planck's constant, *N*_*A*_ is Avogadro's number, η_0_ is the viscosity of the solvent, and *R* is the gas constant. The activation entropy $\Delta S_{2}^{0*}$ and enthalpy $\Delta H_{2}^{0*}$ for different systems could be estimated using the following relations:

(20) $$\begin{array}{l} \Delta S_{2}^{0*}=-\frac{d\left(\Delta \mu_{2}^{0*}\right)}{dT} \end{array}$$

(21) $$\begin{array}{l} \Delta H_{2}^{0*}=\Delta \mu_{2}^{0*}+T\Delta S_{2}^{0*} \end{array}$$

The calculated values of activation free energies, entropies, and enthalpies are listed in Table 6. According to the reported values in Table 6, $\Delta \mu_{2}^{0*}$ values are positive and also larger than $\Delta \mu_{1}^{0*}$ for all the studied systems, which signify the structure-maker ability of [C_2_mim]Cl in aqueous electrolyte solutions and are therefore supporting our conclusions through Hepler's constant and *dB*/*dT*. Larger $\Delta \mu_{2}^{0*}$ values demonstrated the presence of stronger ion–solvent interactions as well. Indeed, the formation of a transition state occurred, followed by the deformation of the intermolecular forces in the solvent structure. Similar results were reported for the amino acids in aqueous salbutamol sulfate solution and glycine in aqueous transition metal chloride solutions (Mishra and Gautam, 2001; Rajagopal and Jayabalakrishnan, 2009).

**Table 6:** The activation free energy of solvent, solute, entropy, and enthalpy for the studied systems at different temperatures.

**T/K**	$\Delta \mu_{1}^{0*}$ (kJ·mol^**−1**^)	$\Delta \mu_{2}^{0*}$ (kJ·mol^**−1**^)	$-\text{T}.\Delta {\text{S}}_{2}^{0*}$ (kJ·mol^**−1**^)	$\Delta {\text{H}}_{2}^{0*}$ (kJ mol^**−1**^)
**[C**_**2**_**mim]Cl** **+** **H**_**2**_**O** **+** **KCl (*****w**_***s***_* **=** **0.11)**				
298.15	9.22	77.92	13.62	64.30
303.15	9.12	77.60	13.85	63.75
308.15	9.05	77.38	14.07	63.31
313.15	8.98	77.20	14.30	62.90
318.15	8.93	77.17	14.53	62.64
**[C**_**2**_**mim]Cl** **+** **H**_**2**_**O** **+** **K**_**2**_**CO**_**3**_ **(*****w**_***s***_* **=** **0.11)**				
298.15	9.90	83.10	29.16	53.94
303.15	9.81	83.05	29.65	53.40
308.15	9.72	81.65	30.14	51.51
313.15	9.66	81.47	30.63	50.84
318.15	9.59	81.36	31.11	50.25
**[C**_**2**_**mim]Cl** **+** **H**_**2**_**O** **+** **K**_**3**_**PO**_**4**_ **(*****w**_***s***_* **=** **0.11)**				
298.15	10.00	90.72	47.60	43.12
303.15	9.91	89.54	48.40	41.15
308.15	9.83	88.14	49.20	38.94
313.15	9.74	87.90	49.99	37.91
318.15	9.67	87.55	50.79	36.76

Based on the transition state theory, each solvent molecule in 1 mol of the solution must pass across the transition state and interact with solute molecules (more/less strongly) (Feakins et al., 1993). The activation free energy ($\Delta \mu_{2}^{0*}$) incorporates the free energy of transfer of the solute from base state to the transition state $\Delta G_{2}^{0}\left(1\to 1^{\prime }\right)$ along with the free energy of solute across its viscous transition state $\left[\Delta G_{2}^{0}\left(2\to 2^{\prime }\right)=\;\Delta \mu_{1}^{0*}\right]$. $\Delta G_{2}^{0}\left(1\to 1^{\prime }\right)$ is calculated using Equation (22) (Feakins et al., 1993; Yan et al., 2002).

(22) $$\begin{array}{l} \Delta G_{2}^{0}\left(1\to 1^{\prime }\right)=\Delta \mu_{2}^{0*}-\Delta \mu_{1}^{0*} \end{array}$$

The obtained values for $\Delta G_{2}^{0}\left(1\to 1^{\prime }\right)$ can be found in Table 7, and they are for all studied systems positive and larger than $\Delta \mu_{1}^{0*}$. This realization revealed that for the formation of a transition state, more solute–solvent bonds should be broken; in other words, the formation of the transition state is less favored (Mishra and Gautam, 2001; Yan et al., 2002; Rajagopal and Jayabalakrishnan, 2009).

**Table 7:** Thermodynamic activation parameter transfer of [C_2_mim]Cl from water to aqueous electrolyte solutions (*w*_*s*_ = 0.11).

	$\Delta {\text{G}}_{2}^{0}\left(1\to 1'\right)$**/kJ·mol**^****−1****^				
**System**	**298.15 K**	**303.15 K**	**308.15 K**	**313.15 K**	**318.15 K**
[C_2_mim]Cl + H_2_O + KCl	68.70	68.48	68.33	68.22	68.24
[C_2_mim]Cl + H_2_O + K_2_CO_3_	73.20	73.24	71.93	71.81	71.77
[C_2_mim]Cl + H_2_O + K_3_PO_4_	80.72	79.63	78.31	78.16	77.88

According to Table 6, $\Delta H_{2}^{0*}$ values are positive for all systems, which means that the achievement of the transition state for viscous flow has occurred along with bond breaking and order decrease. These results are in good agreement with conclusions drawn from apparent molar volume and viscosity, so the results confirm each other.

The temperature dependency of the viscosity was fitted with the Vogel–Fulcher–Tammann (VFT) equation, which is capable of describing the relation between temperature and dynamic viscosity, at constant molar composition, and can be expressed as follows (Verdía et al., 2014; González et al., 2015; Yang et al., 2017; Cai et al., 2020):

(23) $$\begin{array}{l} \eta =A\left[\exp\left(\frac{B}{T-T_{0}}\right)\right] \end{array}$$

**Figure 5:**
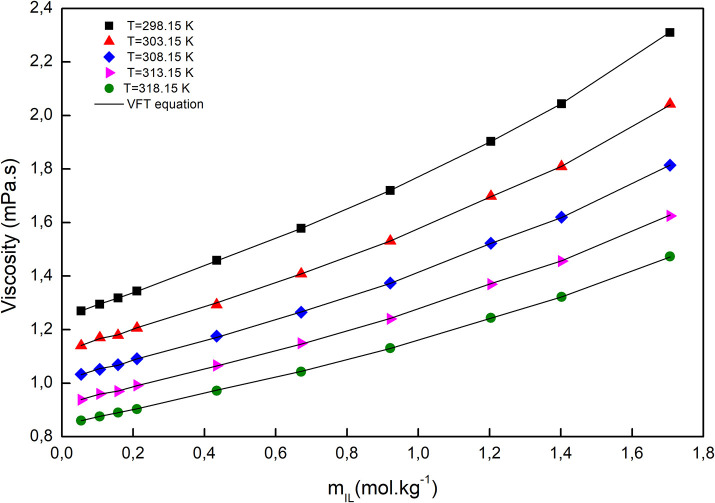
The viscosity of [C_2_mim]Cl + H_2_O + K_3_PO_4_ as a function of [C_2_mim]Cl molality at different temperatures.

*A, B*, and *T*_0_, which are adjustable parameters along with the correlation coefficients (*R*^2^), are given in Supplementary Table 3. As depicted in Figure 5, the viscosity of a solution, at a fixed molality, decreased with increasing temperature. Since the viscosity of a liquid is a measure of the cohesion of the molecules, it is sensitive to temperature. Consequently, the higher the temperature, the stronger the molecules' vibrations, the smaller the molecules' cohesion, and consequently, the lower the viscosity. The viscosity of all solutions increased by increasing molality, due to the smaller distances between anions and cations, so electrostatic interactions became stronger and resulted in increased viscosity.

### FTIR Analysis

**Figure 6:**
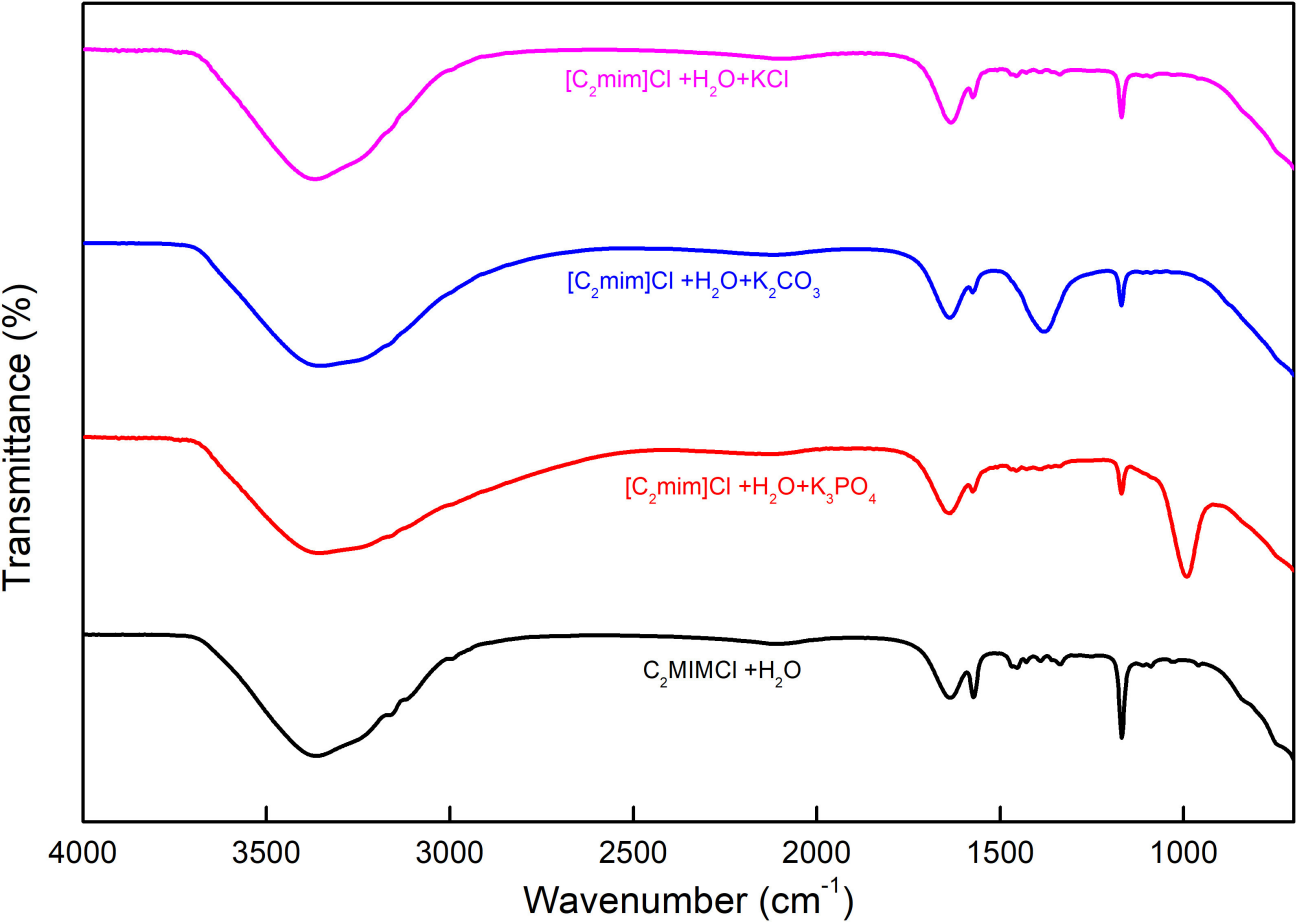
FTIR spectra of [C_2_mim]Cl + H_2_O and [C_2_mim]Cl + H_2_O + electrolyte.

For the structure determination of all kinds of compounds including inorganic or organic, FTIR spectroscopy is the most important characterization technique and can be employed. This technique can be applied for both a single compound and a mixture of compounds in a powdered, solid, liquid, or paste state. The FTIR method supplies insight into the molecular interactions that take place in the systems, based on the vibrations of the atoms of a molecule. The FTIR apparatus sends the infrared radiations toward the sample, and some of them pass through the sample whereas the other part is absorbed by the molecules of the sample. The absorbed radiations are transformed into vibrational or rotational energy by the molecules of the sample. The resulting spectra represent the fingerprint of the molecule. The FTIR spectra of [C_2_mim]Cl + H_2_O and [C_2_mim]Cl + H_2_O + electrolytes were recorded in the wavenumber (4.000–400 cm^−1^), and the values are depicted in Figure 6.

The aqueous solution of [C_2_mim]Cl gives rise to major peaks at 1.165 cm^−1^, corresponding to the stretching vibration of the amine group (C–N), 1.350–1.454 cm^−1^ bending of the C–H, 1.575 cm^−1^ stretching vibration of C=C, and 1.633 cm^−1^ stretching vibration of N=C. The broad peak corresponding to the O–H stretching band for water appears at 3.356 cm^−1^ (Seethalakshmi et al., 2012).

As shown in Figure 6, the spectrum of [C_2_mim]Cl + H_2_O + K_3_PO_4_ shows a band around 991 cm^−1^, which is related to the asymmetric stretching P–O in aqueous ${\text{PO}}_{4}^{3-}$ ions besides the earlier-mentioned peaks for [C_2_mim]Cl (Jastrzebski et al., 2011; Zhang et al., 2018). Furthermore, the spectrum of [C_2_mim]Cl + H_2_O + K_2_CO_3_ comprises characteristic peaks for [C_2_mim]Cl as well as a peak at 1,381 cm^−1^ that belongs to the asymmetric stretching of CO_2_ in a carbonate structure (Toops et al., 2005; Liu et al., 2017).

According to Figure 6, there is no significant difference between the spectra of aqueous [C_2_mim]Cl and [C_2_mim]Cl + H_2_O + KCl due to the dissociation of KCl in the aqueous medium, which does not show any absorbance to the infrared radiations (Max and Chapados, 1999).

The broad O–H peaks in all the studied samples arise from the water molecules in the solution around [C_2_mim]Cl and also K_3_PO_4_, K_2_CO_3_, and KCl molecules. However, a small change in FTIR wavenumber of O–H peak in different systems may be interpreted by some structural change or possible interactions between [C_2_mim]Cl and electrolyte molecules. A similar behavior was reported recently for the IL + water + amino acid systems (Kumar and Sharma, 2020; Kumar et al., 2020).

### Thermal Properties

**Figure 7:**
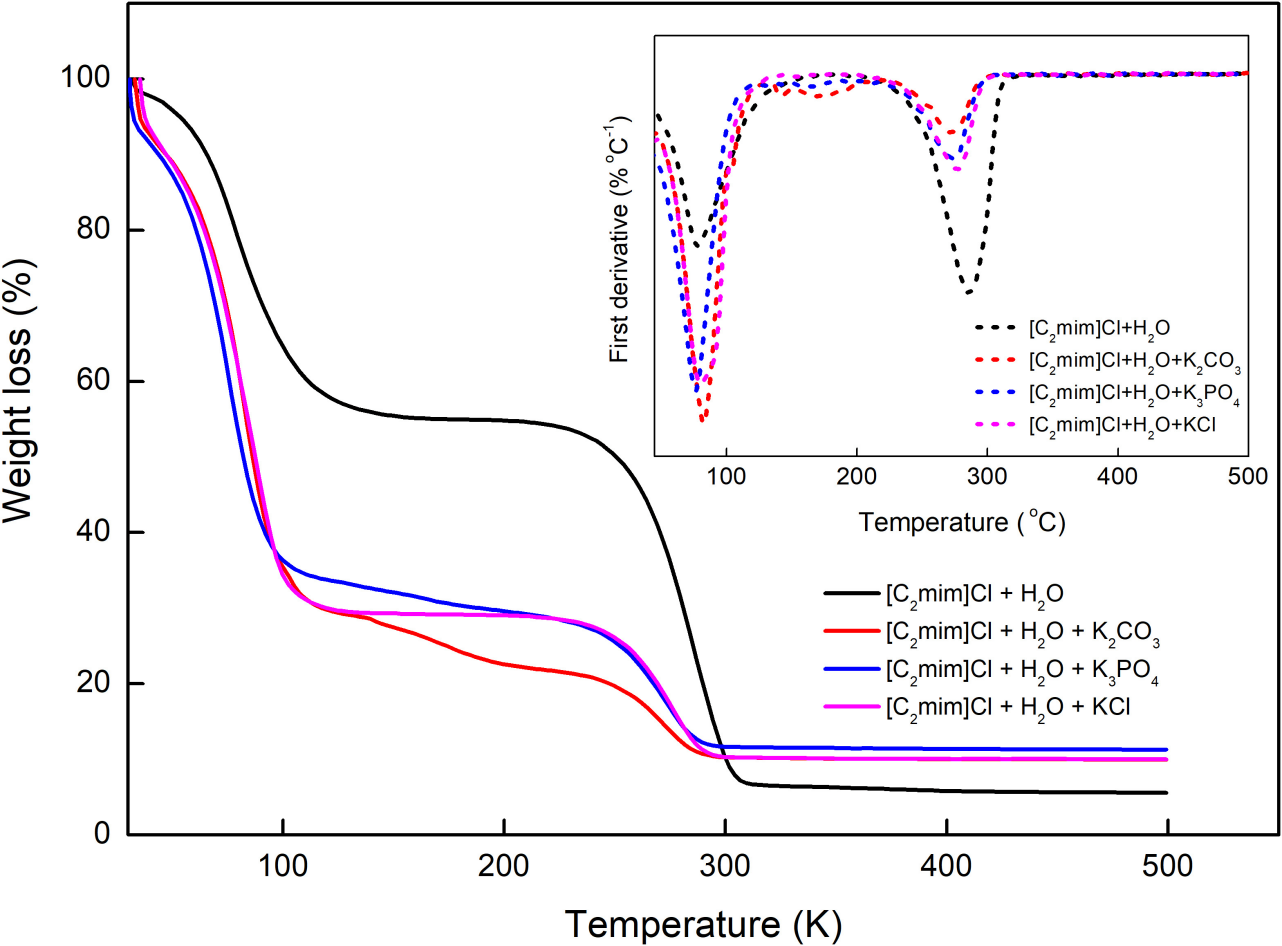
TGA and DTA curve of [C_2_mim]Cl + H_2_O and [C_2_mim]Cl + H_2_O + electrolyte.

The thermal behavior of [C_2_mim]Cl in water and aqueous electrolyte solutions was also investigated, and the results are presented in Figure 7. According to TGA–DTA diagrams, the studied systems undergo a two-step thermal decomposition process (Efimova et al., 2015). The first step occurred around 70–80°C, which can be attributed to the dehydration of the samples. The decomposition of [C_2_mim]Cl takes place at 286.7°C, and the addition of different electrolytes shifted the decomposition of [C_2_mim]Cl to lower temperatures 271.7, 274.2, and 276.7, respectively, for K_3_PO_4_, K_2_CO_3_, and KCl. Therefore, increasing the charge of anions resulted in the decomposition of IL at lower temperatures and decreased the thermal stability of IL more.

## Conclusions

The density, speed of sound, and viscosity of [C_2_mim]Cl in water and aqueous KCl, K_2_CO_3_, and K_3_PO_4_ (*w*_*s*_ = 0.11) solutions were measured at *T* = 298.15–318.15 K. From the experimental data, the apparent molar volume, apparent isentropic compressibility, and relative viscosity have been determined. The limiting apparent molar volume and isentropic compressibility along with viscosity *B*-coefficient were calculated as well, which were utilized to evaluate the corresponding transfer parameters for [C_2_mim]Cl from water to aqueous electrolyte solution. The obtained result exhibited a negative transfer volume of [C_2_mim]Cl from water to the aqueous electrolyte solutions, which decreased by increasing temperature. The isentropic compressibility of all studied systems decreased by increasing the concentration of [C_2_mim]Cl and temperature. The intersection point in isentropic compressibility isotherms may indicate the formation of a clathrate-like structure. Hepler's constant and *dB*/*dT* revealed that [C_2_mim]Cl in pure water and electrolyte solutions acts as a structure maker. The activation free energy $\Delta \mu_{2}^{0*}$ and activation enthalpy $\Delta H_{2}^{0*}\;$values were also calculated, which confirm the structure maker behavior of [C_2_mim]Cl in aqueous electrolyte solutions. The FTIR studies revealed that a change in the wavenumber of the O–H peak in different systems might indicate some structural changes or possible interactions between [C_2_mim]Cl and electrolyte molecules. The temperature dependency of viscosity was satisfactorily fitted to the VFT equation. The viscosity of [C_2_mim]Cl in aqueous electrolyte solutions is larger than that in pure water and increases with increasing IL concentration and decreases with temperature. The TGA revealed that all the studied systems undergo a two-step thermal decomposition process. By the addition of different electrolytes to the aqueous IL solution, the thermal decomposition temperature of IL was shifted to lower temperatures. The higher the anion charge, the lower the decomposition temperature.

## Data Availability Statement

All datasets generated for this study are included in the article/Supplementary Material.

## Author Contributions

SS conducted and organized all the experiments, calculated and analyzed the data, and wrote the manuscript. MZ-M and J-PM supervised the experiments and analysis and edited and reviewed the manuscript. DN performed thermal analysis, analyzed data, and edited and reviewed the manuscript. All the authors contributed to the manuscript and approved the submitted manuscript.

## Conflict of Interest

The authors declare that the research was conducted in the absence of any commercial or financial relationships that could be construed as a potential conflict of interest.
