# Supplementary material for: How Different Electrolytes Can Influence the Aqueous Solution Behavior of 1-Ethyl-3-Methylimidazolium Chloride: A Volumetric, Viscometric, and Infrared Spectroscopy Approach
Source: Front Chem. 2020 Nov 12;8:593786. doi: 10.3389/fchem.2020.593786 (PMC7688583; doi:10.3389/fchem.2020.593786)
Supplement: Supplementary file 1 [file Table_1.docx]

Supplementary Material

**Supplementary Table** **1**. Experimental density/(kg·m^-3^), speed of sound/(m·s^-1^) of [C_2_mim]Cl in water and studied electrolyte solutions

| m_IL_ (mol·kg^-1^) | ρ | | u | | ρ | | u | | ρ | | u | | ρ | | u | ρ | u |
| --- | --- | --- | --- | --- | --- | --- | --- | --- | --- | --- | --- | --- | --- | --- | --- | --- | --- |
|  | 298.15 K | | | | 303.15 K | | | | 3083.15 K | | | | 313.15 K | | | 318.15 K | |
| [C_2_mim]Cl + H_2_O | | | | | | | | | | | | | | | | | |
| 0.0338 | 997.60 | 1500.59 | | 996.19 | | 1512.76 | | 994.56 | | 1523.26 | | 992.73 | | 1532.18 | | 990.70 | 1539.60 |
| 0.0709 | 998.21 | 1504.70 | | 996.78 | | 1516.56 | | 995.15 | | 1526.83 | | 993.30 | | 1535.54 | | 991.25 | 1542.76 |
| 0.1419 | 999.36 | 1512.06 | | 997.92 | | 1523.56 | | 996.26 | | 1533.39 | | 994.39 | | 1541.77 | | 992.30 | 1548.70 |
| 0.2114 | 1000.48 | | 1519.36 | | 999.02 | | 1530.38 | | 997.33 | | 1539.80 | | 995.45 | | 1547.77 | 993.33 | 1554.31 |
| 0.2893 | 1001.73 | | 1527.36 | | 1000.24 | | 1537.87 | | 998.53 | | 1546.88 | | 996.63 | | 1554.46 | 994.48 | 1560.65 |
| 0.3603 | 1002.85 | | 1534.43 | | 1001.34 | | 1544.54 | | 999.61 | | 1553.18 | | 997.69 | | 1560.40 | 995.52 | 1566.26 |
| 0.4347 | 1004.02 | | 1541.82 | | 1002.48 | | 1551.51 | | 1000.73 | | 1559.73 | | 998.80 | | 1566.59 | 996.62 | 1572.11 |
| 0.5152 | 1005.27 | | 1549.75 | | 1003.70 | | 1558.96 | | 1001.94 | | 1566.75 | | 999.97 | | 1573.21 | 997.81 | 1578.38 |
| 0.5957 | 1006.52 | | 1557.49 | | 1004.92 | | 1566.25 | | 1003.14 | | 1573.62 | | 1001.15 | | 1579.70 | 998.97 | 1584.52 |
| 0.6775 | 1007.76 | | 1565.23 | | 1006.14 | | 1573.54 | | 1004.32 | | 1580.51 | | 1002.34 | | 1586.21 | 1000.17 | 1590.66 |
| 0.7564 | 1008.97 | | 1572.56 | | 1007.31 | | 1580.53 | | 1005.48 | | 1587.16 | | 1003.48 | | 1592.47 | 1001.32 | 1596.55 |
| [C_2_mim]Cl + KCl (w_s_ = 0.11) + H_2_O | | | | | | | | | | | | | | | | | |
| 0.0528 | 1068.25 | | 1580.75 | | 1066.38 | | 1589.84 | | 1064.37 | | 1597.93 | | 1062.24 | | 1604.29 | 1059.98 | 1609.74 |
| 0.1039 | 1068.74 | | 1586.47 | | 1066.87 | | 1595.21 | | 1064.84 | | 1602.25 | | 1062.69 | | 1609.12 | 1060.42 | 1614.25 |
| 0.1590 | 1069.30 | | 1592.45 | | 1067.41 | | 1600.86 | | 1065.37 | | 1607.69 | | 1063.20 | | 1614.22 | 1060.93 | 1618.98 |
| 0.2087 | 1069.83 | | 1597.74 | | 1067.92 | | 1605.89 | | 1065.87 | | 1614.75 | | 1063.72 | | 1618.68 | 1061.42 | 1623.14 |
| 0.4345 | 1072.53 | | 1621.02 | | 1070.53 | | 1627.85 | | 1068.45 | | 1633.59 | | 1066.22 | | 1638.35 | 1063.89 | 1641.40 |
| 0.6771 | 1075.88 | | 1644.68 | | 1073.77 | | 1650.20 | | 1071.72 | | 1654.71 | | 1069.27 | | 1658.25 | 1066.95 | 1659.69 |
| 0.8903 | 1079.19 | | 1664.22 | | 1076.94 | | 1668.86 | | 1074.91 | | 1669.95 | | 1072.55 | | 1674.31 | 1070.04 | 1674.38 |
| 1.2021 | 1084.72 | | 1691.49 | | 1082.27 | | 1694.57 | | 1080.24 | | 1697.60 | | 1077.59 | | 1696.70 | 1075.19 | 1694.20 |
| 1.3971 | 1088.44 | | 1707.80 | | 1086.04 | | 1709.55 | | 1083.72 | | 1711.50 | | 1081.32 | | 1709.68 | 1078.82 | 1705.60 |
| 1.7150 | 1094.82 | | 1732.75 | | 1092.53 | | 1733.25 | | 1090.31 | | 1733.79 | | 1087.70 | | 1729.25 | 1085.15 | 1723.20 |
| [C_2_mim]Cl + K_2_CO_3_ (w_s_ = 0.11) + H_2_O | | | | | | | | | | | | | | | | | |
| 0.0521 | 1097.53 | | 1636.67 | | 1095.49 | | 1644.75 | | 1093.32 | | 1651.36 | | 1091.04 | | 1656.93 | 1088.65 | 1661.21 |
| 0.1039 | 1097.90 | | 1642.35 | | 1095.85 | | 1650.04 | | 1093.65 | | 1656.31 | | 1091.37 | | 1661.71 | 1088.95 | 1665.76 |
| 0.1594 | 1098.34 | | 1648.15 | | 1096.25 | | 1655.64 | | 1094.04 | | 1661.46 | | 1091.76 | | 1666.63 | 1089.35 | 1670.41 |
| 0.2112 | 1098.78 | | 1653.53 | | 1096.69 | | 1660.59 | | 1094.43 | | 1666.28 | | 1092.13 | | 1671.08 | 1089.73 | 1674.60 |
| 0.4407 | 1101.06 | | 1676.35 | | 1098.87 | | 1682.01 | | 1096.53 | | 1686.57 | | 1094.22 | | 1690.07 | 1091.75 | 1692.53 |
| 0.6748 | 1103.79 | | 1698.48 | | 1101.68 | | 1702.50 | | 1099.30 | | 1705.62 | | 1096.91 | | 1708.02 | 1094.43 | 1709.08 |
| 0.9264 | 1107.54 | | 1720.10 | | 1105.13 | | 1723.20 | | 1102.70 | | 1725.37 | | 1100.27 | | 1726.64 | 1097.75 | 1727.03 |
| 1.2063 | 1111.94 | | 1743.20 | | 1109.76 | | 1744.87 | | 1107.56 | | 1744.68 | | 1104.77 | | 1746.81 | 1102.40 | 1744.94 |
| 1.4556 | 1116.71 | | 1762.76 | | 1114.49 | | 1764.23 | | 1112.10 | | 1762.92 | | 1109.37 | | 1763.87 | 1106.90 | 1761.10 |
| 1.7018 | 1121.83 | | 1781.41 | | 1119.68 | | 1783.99 | | 1117.20 | | 1782.86 | | 1114.37 | | 1781.04 | 1111.72 | 1778.55 |
| [C_2_mim]Cl + K_3_PO_4_ (w_s_ = 0.11) + H_2_O | | | | | | | | | | | | | | | | | |
| 0.0540 | 1109.05 | | 1620.82 | | 1106.94 | | 1629.09 | | 1104.70 | | 1636.06 | | 1102.35 | | 1641.79 | 1099.88 | 1646.37 |
| 0.1061 | 1109.43 | | 1628.90 | | 1107.32 | | 1636.72 | | 1105.06 | | 1643.30 | | 1102.71 | | 1648.64 | 1100.23 | 1652.85 |
| 0.1575 | 1109.84 | | 1633.02 | | 1107.71 | | 1640.56 | | 1105.47 | | 1646.85 | | 1103.09 | | 1651.97 | 1100.61 | 1655.96 |
| 0.2107 | 1110.31 | | 1639.60 | | 1108.17 | | 1646.75 | | 1105.91 | | 1652.70 | | 1103.52 | | 1657.47 | 1101.03 | 1661.13 |
| 0.4344 | 1112.61 | | 1664.83 | | 1110.44 | | 1670.49 | | 1108.12 | | 1675.05 | | 1105.74 | | 1678.54 | 1103.27 | 1681.02 |
| 0.6713 | 1115.74 | | 1686.32 | | 1113.40 | | 1690.65 | | 1111.04 | | 1693.98 | | 1108.65 | | 1696.31 | 1106.23 | 1697.71 |
| 0.9214 | 1119.66 | | 1709.05 | | 1117.29 | | 1712.05 | | 1114.88 | | 1714.12 | | 1112.38 | | 1715.28 | 1109.90 | 1715.58 |
| 1.2036 | 1124.70 | | 1735.25 | | 1122.37 | | 1736.79 | | 1120.08 | | 1737.49 | | 1117.24 | | 1737.38 | 1114.72 | 1736.49 |
| 1.4017 | 1128.73 | | 1751.14 | | 1126.47 | | 1751.77 | | 1123.96 | | 1751.62 | | 1121.37 | | 1750.71 | 1118.70 | 1749.06 |
| 1.7062 | 1135.68 | | 1777.58 | | 1133.19 | | 1776.85 | | 1130.61 | | 1775.39 | | 1127.95 | | 1773.26 | 1125.22 | 1770.46 |
