# Supplementary material for: How Different Electrolytes Can Influence the Aqueous Solution Behavior of 1-Ethyl-3-Methylimidazolium Chloride: A Volumetric, Viscometric, and Infrared Spectroscopy Approach
Source: Front Chem. 2020 Nov 12;8:593786. doi: 10.3389/fchem.2020.593786 (PMC7688583; doi:10.3389/fchem.2020.593786)
Supplement: Supplementary file 2 [file Table_2.docx]

Supplementary Material

**Supplementary Table** **2**. Experimental viscosity/(mPa⋅s) data as a function of IL’s molarity/(mol⋅dm^-3^) for the studied systems

| T / K | | | | | | | | | | | | | |
| --- | --- | --- | --- | --- | --- | --- | --- | --- | --- | --- | --- | --- | --- |
| 298.15 | | 303.15 | | | 308.15 | | | | 313.15 | | | 318.15 | |
| *c* | *η* | | *c* | *η* | | *c* | *η* | *c* | | *η* | *c* | | *η* |
| [C_2_mim]Cl + H_2_O | | | | | | | | | | | | | |
| 0.0700 | 0.905 | | 0.0699 | 0.811 | | 0.0698 | 0.732 | 0.0697 | | 0.665 | 0.0696 | | 0.605 |
| 0.1389 | 0.921 | | 0.1387 | 0.825 | | 0.1385 | 0.743 | 0.1382 | | 0.674 | 0.1379 | | 0.618 |
| 0.2051 | 0.939 | | 0.2048 | 0.840 | | 0.2045 | 0.757 | 0.2041 | | 0.688 | 0.2037 | | 0.630 |
| 0.2780 | 0.954 | | 0.2776 | 0.853 | | 0.2771 | 0.770 | 0.2766 | | 0.697 | 0.2760 | | 0.638 |
| 0.3432 | 0.972 | | 0.3427 | 0.869 | | 0.3421 | 0.783 | 0.3414 | | 0.711 | 0.3407 | | 0.649 |
| 0.4103 | 0.992 | | 0.4097 | 0.885 | | 0.4090 | 0.796 | 0.4082 | | 0.723 | 0.4073 | | 0.662 |
| 0.4815 | 1.014 | | 0.4808 | 0.901 | | 0.4799 | 0.815 | 0.4790 | | 0.737 | 0.4780 | | 0.675 |
| 0.5514 | 1.036 | | 0.5505 | 0.924 | | 0.5496 | 0.829 | 0.5485 | | 0.752 | 0.5473 | | 0.688 |
| 0.6211 | 1.049 | | 0.6201 | 0.937 | | 0.6189 | 0.845 | 0.6177 | | 0.765 | 0.6164 | | 0.701 |
| 0.6870 | 1.073 | | 0.6859 | 0.957 | | 0.6846 | 0.861 | 0.6833 | | 0.781 | 0.6818 | | 0.712 |
| 0.0336 | 0.896 | | 0.0335 | 0.801 | | 0.0335 | 0.721 | 0.0334 | | 0.658 | 0.0333 | | 0.600 |
| [C_2_mim]Cl + H_2_O + KCl (*w*_s_ = 0. 11) | | | | | | | | | | | | | |
| 0.056 | 0.898 | | 0.0559 | 0.815 | | 0.0558 | 0.745 | 0.0557 | | 0.683 | 0.0555 | | 0.632 |
| 0.1094 | 0.914 | | 0.1092 | 0.832 | | 0.109 | 0.757 | 0.1088 | | 0.697 | 0.1085 | | 0.643 |
| 0.1661 | 0.931 | | 0.1659 | 0.844 | | 0.1655 | 0.771 | 0.1652 | | 0.707 | 0.1648 | | 0.652 |
| 0.2166 | 0.944 | | 0.2163 | 0.856 | | 0.2158 | 0.780 | 0.2154 | | 0.715 | 0.2149 | | 0.663 |
| 0.4381 | 1.013 | | 0.4373 | 0.916 | | 0.4364 | 0.835 | 0.4355 | | 0.763 | 0.4346 | | 0.706 |
| 0.6627 | 1.089 | | 0.6614 | 0.983 | | 0.6601 | 0.893 | 0.6586 | | 0.821 | 0.6572 | | 0.753 |
| 0.8499 | 1.155 | | 0.8481 | 1.049 | | 0.8465 | 0.951 | 0.8446 | | 0.869 | 0.8427 | | 0.800 |
| 1.1086 | 1.271 | | 1.1061 | 1.150 | | 1.1040 | 1.041 | 1.1013 | | 0.954 | 1.0988 | | 0.875 |
| 1.2621 | 1.350 | | 1.2593 | 1.217 | | 1.2567 | 1.103 | 1.2539 | | 1.006 | 1.2510 | | 0.924 |
| 1.5004 | 1.480 | | 1.4972 | 1.316 | | 1.4942 | 1.190 | 1.4906 | | 1.080 | 1.4871 | | 0.990 |
| [C_2_mim]Cl + H_2_O + K_2_CO_3_ (*w*_s_ = 0. 11) | | | | | | | | | | | | | |
| 0.0567 | 1.183 | | 0.0566 | 1.068 | | 0.0565 | 0.970 | 0.0564 | | 0.887 | 0.0563 | | 0.808 |
| 0.1124 | 1.201 | | 0.1121 | 1.083 | | 0.1119 | 0.980 | 0.1117 | | 0.894 | 0.1114 | | 0.819 |
| 0.1711 | 1.222 | | 0.1708 | 1.100 | | 0.1704 | 0.995 | 0.1701 | | 0.911 | 0.1697 | | 0.832 |
| 0.2251 | 1.244 | | 0.2247 | 1.117 | | 0.2242 | 1.011 | 0.2237 | | 0.924 | 0.2232 | | 0.847 |
| 0.4558 | 1.345 | | 0.4549 | 1.206 | | 0.4539 | 1.088 | 0.4530 | | 0.994 | 0.4519 | | 0.907 |
| 0.6778 | 1.447 | | 0.6765 | 1.298 | | 0.6750 | 1.171 | 0.6736 | | 1.066 | 0.6720 | | 0.971 |
| 0.9033 | 1.56 | | 0.9014 | 1.400 | | 0.8994 | 1.258 | 0.8974 | | 1.143 | 0.8953 | | 1.045 |
| 1.1398 | 1.727 | | 1.1375 | 1.549 | | 1.1353 | 1.386 | 1.1324 | | 1.251 | 1.1300 | | 1.142 |
| 1.3396 | 1.886 | | 1.3369 | 1.685 | | 1.3341 | 1.472 | 1.3308 | | 1.364 | 1.3278 | | 1.238 |
| 1.5279 | 2.017 | | 1.5250 | 1.793 | | 1.5216 | 1.605 | 1.5177 | | 1.450 | 1.5141 | | 1.315 |
| [C_2_mim]Cl + H_2_O + K_3_PO_4_ (*w*_s_ = 0. 11) | | | | | | | | | | | | | |
| 0.0594 | 1.270 | | 0.0593 | 1.140 | | 0.0592 | 1.033 | 0.0591 | | 0.938 | 0.0589 | | 0.860 |
| 0.1159 | 1.295 | | 0.1157 | 1.170 | | 0.1155 | 1.052 | 0.1152 | | 0.960 | 0.1149 | | 0.876 |
| 0.1709 | 1.318 | | 0.1705 | 1.179 | | 0.1702 | 1.069 | 0.1698 | | 0.970 | 0.1694 | | 0.890 |
| 0.2269 | 1.344 | | 0.2265 | 1.206 | | 0.2260 | 1.091 | 0.2255 | | 0.992 | 0.2250 | | 0.903 |
| 0.4544 | 1.459 | | 0.4535 | 1.293 | | 0.4525 | 1.176 | 0.4516 | | 1.066 | 0.4506 | | 0.972 |
| 0.6819 | 1.578 | | 0.6805 | 1.408 | | 0.6790 | 1.265 | 0.6776 | | 1.149 | 0.6761 | | 1.043 |
| 0.9089 | 1.720 | | 0.9069 | 1.531 | | 0.9050 | 1.374 | 0.9030 | | 1.240 | 0.9009 | | 1.131 |
| 1.1506 | 1.903 | | 1.1483 | 1.698 | | 1.1459 | 1.523 | 1.1430 | | 1.370 | 1.1404 | | 1.244 |
| 1.3124 | 2.044 | | 1.3098 | 1.809 | | 1.3069 | 1.620 | 1.3039 | | 1.456 | 1.3007 | | 1.322 |
| 1.5500 | 2.310 | | 1.5466 | 2.042 | | 1.5430 | 1.814 | 1.5394 | | 1.625 | 1.5357 | | 1.473 |
