# Supplementary material for: How Different Electrolytes Can Influence the Aqueous Solution Behavior of 1-Ethyl-3-Methylimidazolium Chloride: A Volumetric, Viscometric, and Infrared Spectroscopy Approach
Source: Front Chem. 2020 Nov 12;8:593786. doi: 10.3389/fchem.2020.593786 (PMC7688583; doi:10.3389/fchem.2020.593786)
Supplement: Supplementary file 3 [file Table_3.docx]

Supplementary Material

**Supplementary Table** **3**. Parameters of VFT Equation for studied systems at different [C_2_mim]Cl molalities

| m_IL_  (mol·kg^-1^) | A (mPa·s) | B (K) | T_0_ (K) | R^2^ |
| --- | --- | --- | --- | --- |
| [C_2_mim]Cl + H_2_O | | | | |
| 0.0338 | 0.058 | 322.1 | 180.8 | 0.9893 |
| 0.0709 | 0.018 | 681.0 | 123.7 | 0.9890 |
| 0.1419 | 0.050 | 367.1 | 172.4 | 0.9892 |
| 0.2114 | 0.059 | 327.5 | 179.7 | 0.9890 |
| 0.2893 | 0.043 | 417.2 | 163.9 | 0.9900 |
| 0.3603 | 0.045 | 407.8 | 165.7 | 0.9898 |
| 0.4347 | 0.077 | 271.8 | 191.9 | 0.9877 |
| 0.5152 | 0.080 | 267.6 | 193.0 | 0.9878 |
| 0.5957 | 0.073 | 290.8 | 188.7 | 0.9880 |
| 0.6775 | 0.060 | 347.6 | 176.6 | 0.9890 |
| 0.7564 | 0.054 | 378.4 | 171.9 | 0.9892 |
| [C_2_mim]Cl + H_2_O + KCl (w_s_ = 0.11) | | | | |
| 0.0528 | 0.053 | 398.6 | 157.3 | 0.9910 |
| 0.1039 | 0.042 | 478.1 | 143.1 | 0.9922 |
| 0.1590 | 0.043 | 467.5 | 145.8 | 0.9911 |
| 0.2087 | 0.080 | 293.4 | 179.2 | 0.9904 |
| 0.4345 | 0.073 | 330.4 | 172.7 | 0.9906 |
| 0.6771 | 0.069 | 360.2 | 167.8 | 0.9908 |
| 0.8903 | 0.023 | 748.4 | 106.6 | 0.9930 |
| 1.2021 | 0.045 | 528.6 | 139.6 | 0.9918 |
| 1.3971 | 0.059 | 455.9 | 152.9 | 0.9912 |
| 1.7150 | 0.144 | 223.3 | 202.2 | 0.9868 |
| [C_2_mim]Cl + H_2_O + K_2_CO_3_ (w_s_ = 0.11) | | | | |
| 0.0521 | 0.010 | 1126.0 | 64.3 | 0.9968 |
| 0.1039 | 0.037 | 563.1 | 136.4 | 0.9959 |
| 0.1594 | 0.050 | 471.9 | 150.9 | 0.9956 |
| 0.2112 | 0.086 | 318.5 | 178.9 | 0.9948 |
| 0.4407 | 0.071 | 382.6 | 168.2 | 0.9950 |
| 0.6748 | 0.039 | 579.6 | 137.2 | 0.9957 |
| 0.9264 | 0.059 | 468.0 | 155.2 | 0.9952 |
| 1.2063 | 0.029 | 718.1 | 122.2 | 0.9957 |
| 1.4556 | 0.239 | 160.8 | 220.3 | 0.9916 |
| 1.7018 | 0.065 | 483.5 | 157.4 | 0.9948 |
| [C_2_mim]Cl + H_2_O + K_3_PO_4_ (w_s_ = 0.11) | | | | |
| 0.0540 | 0.051 | 464.2 | 153.6 | 0.9954 |
| 0.1061 | 0.017 | 862.8 | 98.1 | 0.9963 |
| 0.1575 | 0.081 | 339.7 | 176.2 | 0.9947 |
| 0.2107 | 0.023 | 750.5 | 113.3 | 0.9961 |
| 0.4344 | 0.114 | 272.7 | 191.3 | 0.9940 |
| 0.6713 | 0.054 | 484.2 | 154.6 | 0.9951 |
| 0.9214 | 0.076 | 400.4 | 169.7 | 0.9945 |
| 1.2036 | 0.020 | 889.0 | 103.6 | 0.9958 |
| 1.4017 | 0.080 | 417.1 | 169.4 | 0.9943 |
| 1.7062 | 0.074 | 455.5 | 165.8 | 0.9942 |
